# Pelvic congestion syndrome as a differential diagnosis of chronic pelvic pain in women

**DOI:** 10.1016/j.clinsp.2024.100514

**Published:** 2024-10-30

**Authors:** Marcos de Lorenzo Messina, Pedro Puech-Leão, Ricardo dos Santos Simões, Maria Cândida Pinheiro Baracat, José Maria Soares, Edmund Chada Baracat

**Affiliations:** a Gynecology Division, Obstetrics and Gynecology Department, Hospital das Clínicas, Faculdade de Medicina, Universidade de São Paulo (HCFMUSP), São Paulo, SP, Brazil; b Vascular Surgery Division, Surgery Department, Hospital das Clínicas, Faculdade de Medicina, Universidade de São Paulo (HCFMUSP), São Paulo, SP, Brazil

Chronic Pelvic Pain (CPP) is a symptom reported by many women during gynecologist visits and whose cause often remains unknown. According to the Centers for Disease Control and Prevention of the USA (CDC), CPP accounts for nearly 9% of all appointments to gynecologists. Moreover, CPP is responsible for 20%‒30% of all laparoscopies in adults.^1^^,^^2^

Among the differential diagnoses (endometriosis, adenomyosis, pelvic inflammatory disease, adhesions, urological, gastrointestinal, musculoskeletal causes, among others), Pelvic Venous Congestion Syndrome (PVCS) might be perhaps the least considered. However, PVCS may account for one-third of the real causes of CPP in women during the evaluation after all other causes of pain have been excluded.^2^

PVCS is defined as the presence of tortuous or congested ovarian or pelvic veins associated with CPP, in addition to a sensation of pressure and heaviness in the hypogastrium. The pain typically worsens during the premenstrual period, after long periods in the orthostasis position, and after sexual intercourse, whereas its association with varicose veins of the vulva and lower limbs is not uncommon.^3^

The pathophysiology of PVCS is not yet fully understood, but it is known that common causes of varicosities in other regions also play a role in the female pelvic region, such as valve incompetence, blood reflux, and venous engorgement. In addition, ovarian hormonal dysfunction has been suggested to play an important role, since estrogen is known to act as a venous vasodilator.^4^

The anatomy of the venous drainage of the female pelvis may interfere with the pelvic vein dilatation The left ovarian vein flows into the ipsilateral renal vein at a 90° angle, unlike the opening of the right ovarian vein into the inferior vena cava (at an acute angle), which may justify findings of larger venous diameters on the left. Venous compressions leading to stasis such as May-Thurner and/or Nutcracker syndrome also contribute to the etiology of pelvic congestion.^4^

Venography is the gold standard for diagnosing PVCS, as it allows us to identify tortuosities and treat them with angioplasty and/or embolization at the same time. Other less invasive exams are considered in the diagnostic workup, such as angioresonance, angiotomography, and ultrasound, as well as laparoscopy.^2^ Ultrasonography is the least expensive exam for screening and is often one of the first exams requested in the investigation of gynecological conditions that cause CPP.^5^

The diagnosis of pelvic venous congestion syndrome is based on a combination of signs and symptoms, physical examination, and documentation of dilated vessels or pelvic vein incompetence, after excluding other causes. Although the absence of pelvic venous changes helps to exclude PVCS, the presence of these abnormalities is not a diagnostic criterion. Findings of venous dilation on imaging tests must be carefully evaluated and associated with a suggestive clinical picture for diagnostic elucidation.^4^

Clinical treatment is conflicting and lacks scientific evidence. The use of progestogen is discussed in several publications, with medroxyprogesterone acetate showing significant improvement when compared to placebo in up to 9 months of follow-up.^6^^,^^7^ GnRH analog, a blocker of the hypothalamic-pituitary-ovarian axis, can be used for a period of up to 6 months and promotes improvement in CPP at rates higher than medroxyprogesterone acetate. However, the side effects of that drug including depression, anxiety, and sexual dysfunctions can impair the effectiveness in controlling CPP as well as the adherence of treatment.^8^

Regarding surgical treatment, laparoscopic ligation of the gonadal veins was initially suggested as a form of PVCS treatment. However, the complex hemodynamic process involved in PVCS cannot always be resolved by surgical ligation of the ovarian veins. Also, higher recurrence rates of venous disorders were characterized when comparing conventional laparoscopic ligation with endovascular alternatives.

Finally, hysterectomy does not present scientific evidence of improvement in CPP secondary to PVCS and its indication should be discouraged.^9^ According to literature evidence, endovascular techniques such as iliac and renal angioplasty and gonadal vein embolization have been considered the best alternative in the current scientific scenario and further research is necessary in the field.^4^^,^^9^

## Author's contribution

All authors contributed equally to the conceptualization, writing, and reviewing of the present manuscript.

## Declaration of competing interest

The authors declare no conflicts of interest.
